# STCDB4ND: a signal transduction classification database for neurological diseases

**DOI:** 10.1093/database/baaf032

**Published:** 2025-05-02

**Authors:** Boyan Gong, Sida Li, Yifan Chen, Liya Liu, Ralf Hofestädt, Ming Chen

**Affiliations:** Department of Bioinformatics, Institute of Biophysics, College of Life Sciences; Institute of Hematology, Zhejiang University School of Medicine, The First Affiliated Hospital, Zhejiang University, Hangzhou 310058, China; Department of Bioinformatics, Institute of Biophysics, College of Life Sciences; Institute of Hematology, Zhejiang University School of Medicine, The First Affiliated Hospital, Zhejiang University, Hangzhou 310058, China; Department of Bioinformatics, Institute of Biophysics, College of Life Sciences; Institute of Hematology, Zhejiang University School of Medicine, The First Affiliated Hospital, Zhejiang University, Hangzhou 310058, China; Department of Bioinformatics, Institute of Biophysics, College of Life Sciences; Institute of Hematology, Zhejiang University School of Medicine, The First Affiliated Hospital, Zhejiang University, Hangzhou 310058, China; Department of Bioinformatics/Medical Informatics, Faculty of Technology, Bielefeld University, Bielefeld 33501, Germany; Department of Bioinformatics, Institute of Biophysics, College of Life Sciences; Institute of Hematology, Zhejiang University School of Medicine, The First Affiliated Hospital, Zhejiang University, Hangzhou 310058, China; Zhejiang Key Laboratory of Multi-omics Precision Diagnosis and Treatment of Liver Diseases, Zhejiang University, Hangzhou 310058, China

## Abstract

Neurological disorders pose significant global health challenges due to their complex etiology and insufficient understanding of underlying mechanisms. Signal transduction pathways are critical in the pathophysiology of these diseases and have been extensively studied to develop therapeutic interventions. However, existing databases for biological signal pathways often overlook the dynamic interactions between entities within these pathways and lack standardized representations of the signaling processes. To address these limitations, we present STCDB4ND, a specialized database focused on signal transduction pathways associated with neurological diseases. Utilizing the ST classification system, STCDB4ND provides a unified framework for pathway representation, emphasizing interactions and pathway characteristics. The database features advanced visualization tools, network analysis capabilities, and a key factor identification module, enabling researchers to comprehensively study these complex networks. Our analysis of neurological disease-related pathways using STCDB4ND revealed key signaling factors and supported existing findings on pathogenic mechanisms STCDB4ND serves as a valuable resource for advancing the understanding of neurological disease pathways and promoting novel therapeutic approaches. And we believe that STCDB will provide greater convenience for researchers in various fields as we expand the STCDB system’s database in the future.

**Database URL**: https://bis.zju.edu.cn/STCDB

## Introduction

Neurological disorders represent a global public health challenge, affecting individuals across all ages, cultures, and socioeconomic statuses. The complexity of these diseases makes them particularly difficult to study, and as a result, the causes and treatments for many remain not fully understood. In the existing research and treatment methods, cell signal transduction pathways have been confirmed to play an important role in the pathological study of neurological diseases [1]. Many drug design strategies target key nodes within these pathways, highlighting their immense research value. Given the intricate and interwoven nature of biological pathways, thorough analysis and research on these related networks can greatly aid in elucidating the pathogenesis of neurological diseases.

With the ongoing advancement of bioinformatics tools and research, a variety of biological signal pathway databases have emerged [2]. These databases store biological entities and their interactions, aiding researchers in obtaining and analyzing the necessary data. In addition to official institutions focusing on the construction of biological databases, there are also bioinformatics laboratories and biological companies as profit-making organizations that carry out relevant construction. According to different research fields, databases with different focuses have been established, such as cell signaling pathway databases for different species, or specialized signaling pathway databases for signal transduction pathways related to a certain protein family. These biosignaling pathway databases come in different forms, including commercial databases such as Elsevier EmBiology, a platform for exploring biological pathways [3], and QIAGEN Ingenuity Pathway Analysis, a tool for the analysis and visualization of biological pathways and networks [4], which provide high-quality data and robust analytical tools, catering to researchers with demanding precision and integration needs. On the other hand, noncommercial databases like KEGG [5], which contain data written by biologists and reviewed by professionals, and open platforms like WikiPathways [6], which allow users to upload, maintain, and discuss biosignaling pathway data, offer valuable resources to the scientific community. Most databases will give relevant information of each node in the biological signaling pathway network, such as entity name and related genes, directly or through linkage with other databases. Additionally, there are databases like STRING, which focuses on known and predicted protein–protein interactions [7], and ANDSystem, a tool for automatic knowledge extraction and reconstruction of associative gene networks [8], which provide valuable resources for researchers studying biological pathways and their broader connections to molecular networks.

Despite the availability of numerous biological signal pathway databases, many of these resources suffer from certain limitations. The existing databases often attach most of its focus on the entities in the signaling pathway and derive various biological informations based on them. However, the interactions between entities also play an important role in the process of biological signal transduction, which is often neglected when signal pathways are mentioned and analyzed. The classification of the pathways usually accord to the biological function of them, leading to the neglection of the character of the signal transduction pathway itself, which may bring new perspectives and new opportunities for the development of system biology research and precision medicine in the era of “biological big data.” In addition, the representation of signal pathways lacks a unified format, which makes it difficult for researchers to effectively represent these pathways and utilize various downstream analysis tools.

To address these limitations, we present STCDB4ND (https://bis.zju.edu.cn/STCDB), a user-friendly biological signaling transduction pathway database for neurological diseases. This database employs the ST classification system [9] to store and display biological signaling pathways, incorporating the interactions within these pathways. This approach provides a standardized format for representing biosignal pathways and emphasizes the characteristics of the pathways themselves. STCDB4ND is integrated with visualization functions and downstream tools for network analysis, offering a convenient resource for researchers seeking to understand biological signaling transduction pathways related to neurological diseases. By setting a new standard for the representation of these pathways, our database provides researchers with a novel perspective for conducting in-depth research in this field.

## Materials and methods

### ST classification system

The ST classification system [9] assigns a unique four-level code, the ST code, to each entity in the cell signal transduction pathway, similar to the enzyme classification system [10] used by the International Committee on Enzymology. This system allows the interactions between different entities in the pathway to be expressed unambiguously. By converting a signal transduction pathway into a coded string through the ST codes of the interactions, researchers can perform comparison and query operations more easily, facilitating better study of cell signal transduction pathways.

**The coding rules for the ST classification system are as follows**:

- The first digit in the ST code indicates the occurrence of the action relative to the position of the cell.
- The second digit indicates the type of action.
- The third digit combines the characteristics of the two entities involved in the action to further specify the action type.
- The fourth digit identifies the serial number of the action within the same type.

**The specific classifications are shown in**  Table 1.

**Table 1.** Specific classifications of the first two numbers of ST code

ST code	Character of interaction
1.*.*.*	Extracellular signal reception events
1.1.*.*	Physical stimulation of receptors
1.2.*.*	Binding with hormones
1.3.*.*	Binding with non‐growth factor cytokines
1.4.*.*	Binding with growth factors
1.5.*.*	Binding with neuronal receptors
1.6.*.*	Binding with other ligands
2.*.*.*	Plasma membrane transduction events
2.1.*.*	Channel operation
2.2.*.*	Ion channel transduction
2.3.*.*	G‐protein transduction
2.4.*.*	Other Ser/Thr phosphorylation
2.5.*.*	Tyr phosphorylation
2.6.*.*	Cleavage
2.7.*.*	Others
3.*.*.*	Plasma membrane to cytoplasm transduction events
3.1.*.*	Membrane receptor releasing
3.2.*.*	Protein–protein interaction
4.*.*.*	Intracellular signal transduction events
4.1.*.*	Ser/Thr phosphorylation
4.2.*.*	Tyr phosphorylation
4.3.*.*	Other phosphorylation
4.4.*.*	Dephosphorylation
4.5.*.*	Ubiquitination
4.6.*.*	Methylation
4.7.*.*	Deamination
4.8.*.*	Nitrosylation
4.9.*.*	GDP/GTP conversion
4.10.*.*	Dimerization
4.11.*.*	Protein–protein interaction
4.12.*.*	Others
5.*.*.*	Cytoplasm to nucleoplasm transduction events
5.1.*.*	Others
6.*.*.*	Nucleoplasm to nucleoplasm transduction events
6.1.*.*	Binding with nuclear receptor
6.2.*.*	Binding with transcription factor
6.3.*.*	Acetylation
6.4.*.*	Deacetylation
6.5.*.*	Others

### Data source

Data in this database were manually collected from several authoritative databases such as KEGG [5] and Reactome [11]. Each interaction was assigned a unique ST number according to the ST classification system. Protein and small molecule compound data were sourced from Uniprot [12] and CHEMBL [13]. The current database integrates 169 biological signal transduction pathways related to neurological diseases, involving 288 entities and 277 interactions. The selection of these pathways was based on a comprehensive review of multiple pathway databases, including KEGG and Reactome, with a focus on pathways that have reliable literature references.

### Alignment algorithm

The basic idea behind comparing two signal transduction pathways in the ST system is similar to the Smith–Waterman algorithm [14], modified to fit the characteristics of signal pathways. The replacement matrix in our alignment algorithm is generated based on the similarities of the ST numbers in the sequence. The similarity $\sigma \left( {\alpha ,\beta } \right)$ of the two pathways $\alpha \left( {{a_1},{a_2},{a_3},{a_4}} \right)$, $\beta \left( {{b_1},{b_2},{b_3},{b_4}} \right)$ is determined by the following rule:


(1) $$\sigma \left( {\alpha ,\beta } \right) = \left\{ \begin{array}{l}
0,\,{\mathrm{ }}while\,{\mathrm{ a}}1 \ne {\mathrm{b}}1\\
0.25,\,{\mathrm{ }}while\,{\mathrm{ a}}1 = {\mathrm{b}}1\& {\mathrm{a2}} \ne {\mathrm{b2}}\\
{\mathrm{0}}{\mathrm{.5,\, }}while\,{\mathrm{ a1}} = {\mathrm{b1}}\& {\mathrm{a2}} = {\mathrm{b2}}\& {\mathrm{a3}} \ne {\mathrm{b3}}\\
{\mathrm{0}}{\mathrm{.75,\, }}while\,{\mathrm{ a1}} = {\mathrm{b1}}\& {\mathrm{a2}} = {\mathrm{b2}}\& {\mathrm{a3}} = {\mathrm{b3}}\& {\mathrm{a4}} \ne {\mathrm{b4}}\\
{\mathrm{1}},\,{\mathrm{ }}while\,{\mathrm{ a1}} = {\mathrm{b1}}\& {\mathrm{a2}} = {\mathrm{b2}}\& {\mathrm{a3}} = {\mathrm{b3}}\& {\mathrm{a4}} = {\mathrm{b4}}
\end{array} \right.$$

where the gap penalty is set to 0. The similarity score is the maximum matching score of the two pathways, divided by the length of the longer pathway. For pathways $PW1:\left\{ {{\alpha _i}} \right\}$ and $PW2:\left\{ {{\beta _i}} \right\}$, with lengths L1 and L2 respectively, the similarity $f\left( {PW1,PW2} \right)$ is given by:


(2) $$f({\mathrm{PW}}1,{\mathrm{PW}}2) = \frac{{\max \left( {\sum\limits_{{\mathrm{i}} = {\mathrm{1}}}^{{\mathrm{L1}}} {\sigma \left( {\alpha i{\mathrm{ or }}\varepsilon ,\beta j{\mathrm{ or }}\varepsilon } \right)} } \right)}}{{{\mathrm{max(L1,L2)}}}}$$

**Where ε represents the operation to insert a gap element**.

### Key node analysis

To identify key factors in the directed weighted network of cell signal transduction pathways, we referred to the DWNodeRank algorithm [15], which is based on the PageRank algorithm [16]. This algorithm allocates PageRank values according to node weight during transductions, thus introducing the influence of weight in analyzing the key nodes of the network. The element *m_ij_* in the transition matrix M is given by:


(3) $${{\mathrm{m}}_{{\mathrm{ij}}}} = \frac{{{w_{ij}}}}{{\sum\limits_{{v_k} \in M\left( {{v_i}} \right)} {{w_{ik}}} }}$$

where ${w_{ij}}$ is the weight value of the edge from node *j* to node *i*. The importance value $NR\left( {{v_i}} \right)$ of node ${v_i}$ is then calculated as:


(4) $$NR\left( {{v_i}} \right) = d\sum\limits_{{v_j} \in M\left( {{v_i}} \right)} {\frac{{{w_{ij}}}}{{\sum\limits_{{v_k} \in M\left( {{v_i}} \right)} {{w_{ik}}} }}} NR\left( {{v_j}} \right) + \frac{{1 - d}}{n}$$

**Figure 1.**
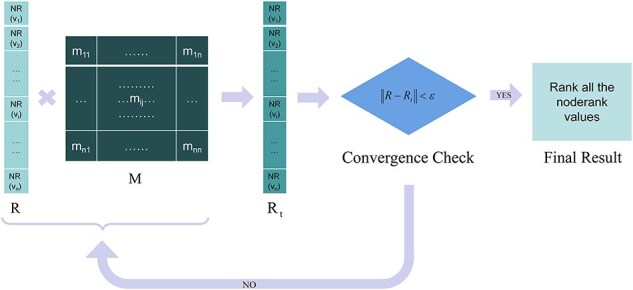
The flowchart of the key factor recognition algorithm, in which M is the transition matrix, R refers to the vector of node-ranks, *d* is damping factor, and ε is the iteration accuracy.

The key node analysis tool in our system uses this method to calculate the NR values of each node in the biosignaling pathway network and sorts these values from high to low to generate key factor recognition results. The algorithm flow is as shown in Fig. 1:

Where ${\mathrm{R}} = \left[ {\begin{array}{*{20}{c}}
{{\mathrm{NR(}}{{\mathrm{v}}_{\mathrm{1}}})}\\
{{\mathrm{NR(}}{{\mathrm{v}}_{\mathrm{2}}})}\\
{\ldots}\\
{{\mathrm{NR(}}{{\mathrm{v}}_{\mathrm{n}}})}
\end{array}} \right]$. The damping factor is set to *d* = 0.85 and iteration accuracy is set to ε = 1e-8 in STCDB system.

### Database construction

**Figure 2.**
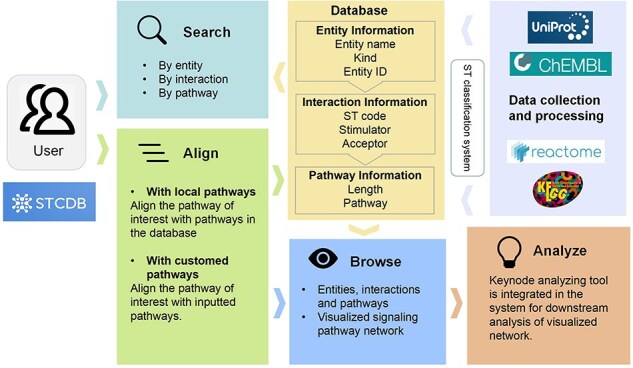
Schematic diagram of the data processing and database structure of STCDB4ND in this study.

STCDB system is accessible for noncommercial use through the website. The server runs on the Linux system Ubuntu 20.04. The frontend was built using Bootstrap4.6 (https://v4.bootcss.com/). The visualization was supported by EChart.js (https://echarts.apache.org/zh/index.html). The backend was supported by Flask2.3.2 (https://flask.palletsprojects.com/en/3.0.x/) and Gunicorn22.0.0 (https://gunicorn.org/). The MySQL8.0.31 database server was implemented for storing and managing all kinds of pathway data (Fig. 2).

## Usage and functions

### Search

**Figure 3.**
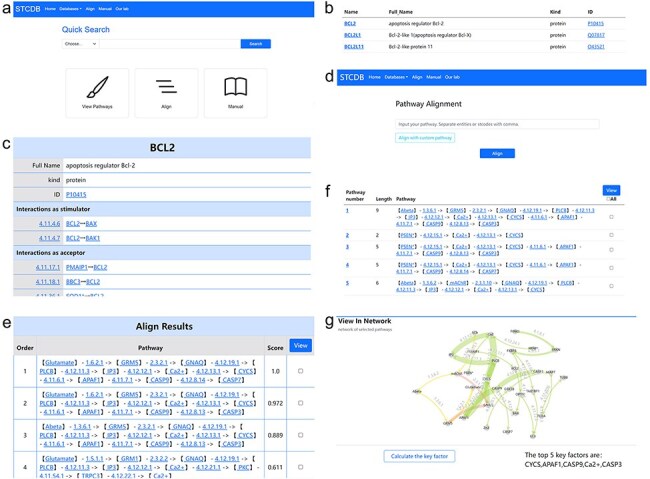
Usage pages in STCDB.

Our database facilitates a flexible and expedient search system that allows users to retrieve biological signaling pathway-related data based on different ranges (Fig. 3A). Users could easily find the search interface on the homepage. The search interface is easily accessible from the homepage. STCDB provides three search modes: entity mode, entity interaction mode, and cell signal transduction pathway mode, each corresponding to distinct sub-database contents. The entity interaction and cell signal transduction pathway modes support search strategies based on both entity name and ST code. Users can access detailed pages for each entry via hyperlinks on the result page (Fig. 3B, C).

### Alignment

The alignment function supports two modes of operation. Users can align the pathway of interest with other signaling pathways in the database, while we also allow users align the pathway of interest with other customized pathways by the user using our alignment page as long as the interactions in the customized pathways are available in our sub database of interactions (Fig. 3D). The pathways can be inputted in the form of either entity strings or ST code strings, using commas as separation. The comparison result page will display the results of each pathway in order of similarity score from high to low (Fig. 3E). The highest similarity score is 1, which indicates that this pathway is the same one as the pathway of interest.

### Visualization of multiple pathway networks

For research involving multiple signaling pathways, the system provides users with the function of pathway network visualization. In the interface of both alignment results and search result page for pathways, users can check the pathways they are interested in and generate the network visualization result of the selected data (Fig. 3E, F). All the entities and interactions are marked with their identification in the network page. The interactions are painted with different colors according to the first digit of its ST number to distinguish the interactions occurred in different relevant positions of the cell (Fig. 3G). Users can interact with the network with their mouse to organize the nodes and lines. It is also available to download the network as still pictures for further research.

### Keynode analysis

To facilitate the analysis of pathway networks, STCDB integrates a key factor analysis tool into the network visualization interface. Users can conveniently identify and analyze key nodes within the network on the same page (Fig. 3G).

## Results

### Whole map of the neuro-disease-related signal pathways

**Figure 4.**
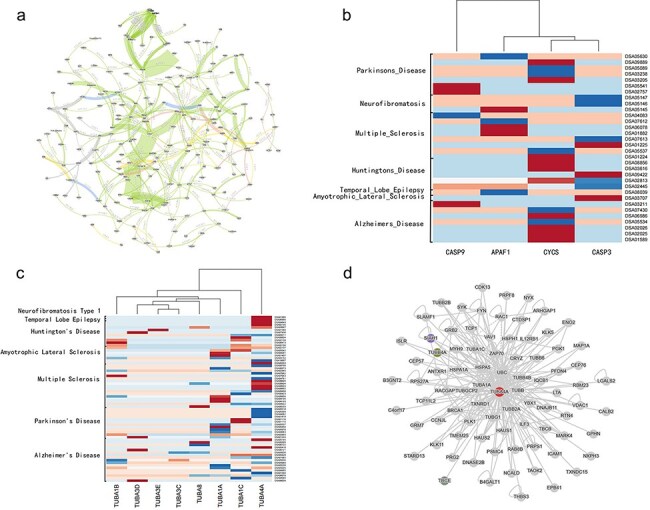
The whole map of the the network of neurological diseases related cell signaling transduction pathways, and figures of heatmap generated from disease data collected from DiSignAtlas website and the PPI network of TUBA4A from NDAtlas website.

We use STCDB to generate a visualized network of all the 169 neurological disease related cell signaling pathways in the system database. The result is as Fig. 4A.

### Key nodes of the neurological disease-related signal pathway network

Using the key factor identification tool of this system to calculate the key factors of the network, the result of top 5 key factors are CYCS, APAF1,CASP9, Ca^2+^,TUBA.

CYCS, APAF1, and CASP9 are important signaling factor in the process of inducing cell apoptosis. This result indicates that in pathways related to neurological diseases, many pathogenic factors ultimately lead to disease by causing abnormal mitochondrial pathway-mediated apoptosis. This finding is consistent with previous research results [17] . We attached transcriptomic data of several neurological diseases collected from DiSignAtlas (http://www.inbirg.com/disignatlas/) to show the differential expression of the related genes [18], and the result is as Fig. 4B that related genes were differentially expressed in multiple datasets (Fig. 4B). The result with high confidence of the CYCS and CASP9 protein from NDAtlas (https://bis.zju.edu.cn/ndatlas/) [19] also shows that these related entities play important roles in neurodegenerative diseases (Supplementary Table 1.).

Ca^2+^ (calcium ion) plays a crucial role in the neurological system, being involved in neurotransmission, signal transduction, and apoptosis. Dysregulation of calcium ions is closely associated with many neurological diseases. Targeting calcium homeostasis presents a promising therapeutic strategy. Calcium channel blockers, calmodulin inhibitors, and drugs regulating calcium homeostasis have shown potential in treating these diseases [20]. Therefore, further research into calcium regulation could lead to novel and effective treatments for various neurodegenerative and neurological disorders, highlighting the critical role of calcium ions in both the pathogenesis and potential therapy of these conditions.

TUBA is a major constituent of microtubules, playing a vital role in cell structure and function. Many neurological diseases are associated with microtubules. According to previous research, damage to microtubules in neuronal axons can lead to the accumulation of pathological proteins, resulting in severe dysfunction of certain neurons. Additionally, there is a correlation between abnormal neuronal activity and changes in microtubule homeostasis, suggesting that alterations in microtubule stability may be a critical factor in the development of neurodegenerative diseases. Microtubule stabilizers have been used to treat chronic neurodegenerative diseases and to intervene in malignant neurological conditions, such as brain tumors [21]. Our identification results provide theoretical support for further research in this direction, reinforcing the belief that microtubule-related studies could be a promising new avenue for the treatment of neurological diseases. We also attached transcriptomic data of several neurological diseases collected from DiSignAtlas [18] to show the differential expression of TUBA-related genes, and the result is as Fig. 4C that related genes were differentially expressed in multiple datasets (Fig. 4C), among which TUBA4A shows the biggest difference comparing with the control group. The PPI network of TUBA4A [19] involves a wide variety of proteins functioning in multiple biological functional processes, reflecting its importance (Fig. 4D).

## Discussion and conclusions

The STCDB4ND database was developed to address critical gaps in the representation and analysis of signal transduction pathways related to neurological diseases. By leveraging the ST classification system, our database standardizes the depiction of these pathways, emphasizing both entity interactions and pathway-specific characteristics. This unified framework enhances the interpretability and comparability of pathways, providing a solid foundation for advanced analyses.

STCDB4ND offers functionalities beyond basic data retrieval, including pathway alignment, multi-pathway visualization, and key factor identification. The alignment feature allows researchers to compare pathways systematically, uncovering similarities and differences that may yield new insights into disease mechanisms Multi-pathway visualization facilitates the exploration of complex networks, reflecting the simultaneous biological activities occurring *in vivo*. Additionally, the key factor analysis tool identifies critical nodes within signaling networks, streamlining the identification of potential therapeutic targets.

Using STCDB4ND, we visualized a comprehensive network of neurological disease-related pathways and identified key signaling factors such as CYCS, APAF1, and CASP9, which are integral to mitochondrial apoptosis pathways. These findings corroborate existing research and highlight the significance of dysregulated apoptosis in the pathogenesis of neurological diseases. The identification of calcium ions (Ca^2+^) and TUBA-related factors further underscores their roles in neural signal transduction and cytoskeletal dynamics, offering promising directions for future research and therapeutic development.

Currently, the STCDB system integrates only cell signal transduction pathways related to human neurological diseases. However, we believe the STCDB system is also applicable to other species and types of cell signal transduction pathways. We aim to expand the STCDB system’s database in the future, incorporating more data to provide greater convenience for researchers in various fields.

To ensure the database remains up-to-date and relevant, we plan to establish a regular update mechanism, conducting comprehensive updates every year. These updates will integrate findings from major biological pathway databases and the latest literature, allowing the inclusion of cutting-edge research discoveries. Additionally, we aim to expand the database over the next five years to include neurological disease-related pathways for more model organisms, such as *Caenorhabditis elegans*, zebrafish, and mice. This will enable researchers to compare differences between various model organisms and humans more effectively.

In the future, we also plan to develop different versions of the database tailored to specific disease types, creating dedicated resources for various categories of diseases. These efforts will significantly enhance the utility and applicability of the STCDB system for researchers in diverse fields.

## Supplementary Material

baaf032_Supp

## Data Availability

The data can be accessed from the databases module of the website.
